# Supplementary figures and images for: The autophagy protein ATG14 safeguards against unscheduled pyroptosis activation to enable embryo transport during early pregnancy
Source: eLife. 2025 Mar 18;13:RP97325. doi: 10.7554/eLife.97325 (PMC11919251; doi:10.7554/eLife.97325)

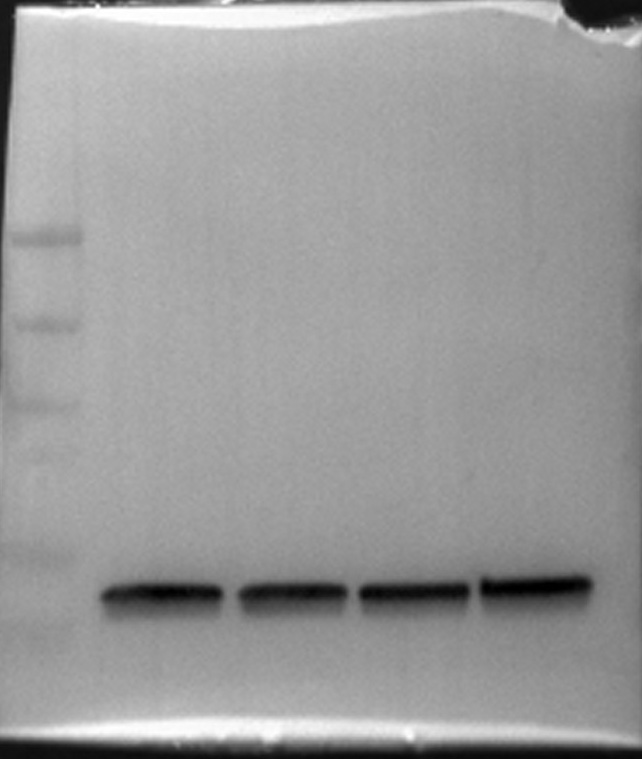

Supplement: Figure 3—figure supplement 2—source data 1. [file elife-97325-fig3-figsupp2-data1.zip › Figure 3-figure supplement 2-source data 1 uncropped original blots/b-actin.jpg]

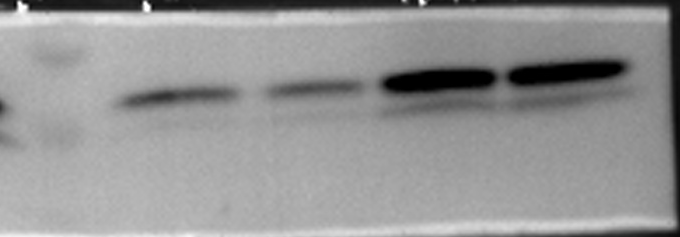

Supplement: Figure 3—figure supplement 2—source data 1. [file elife-97325-fig3-figsupp2-data1.zip › Figure 3-figure supplement 2-source data 1 uncropped original blots/LC3B blot.tif]

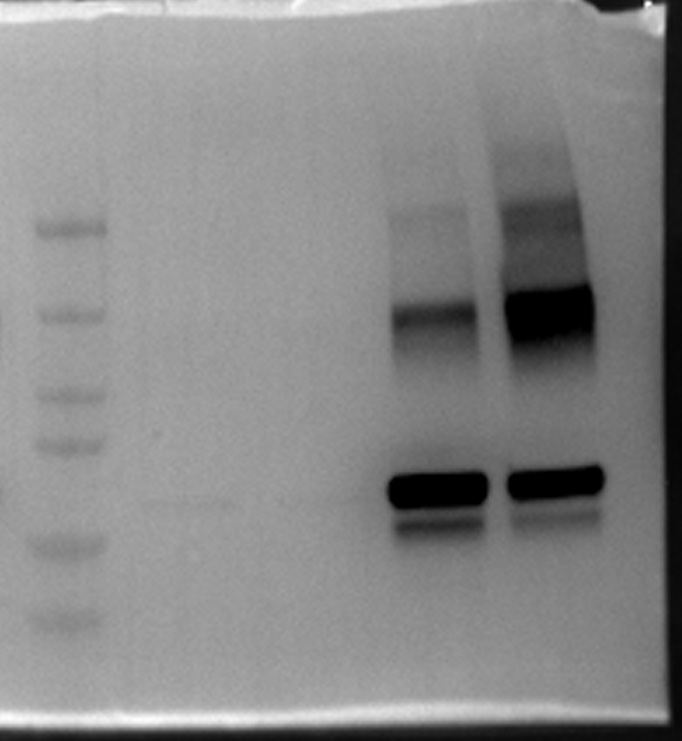

Supplement: Figure 3—figure supplement 2—source data 1. [file elife-97325-fig3-figsupp2-data1.zip › Figure 3-figure supplement 2-source data 1 uncropped original blots/p62.jpg]

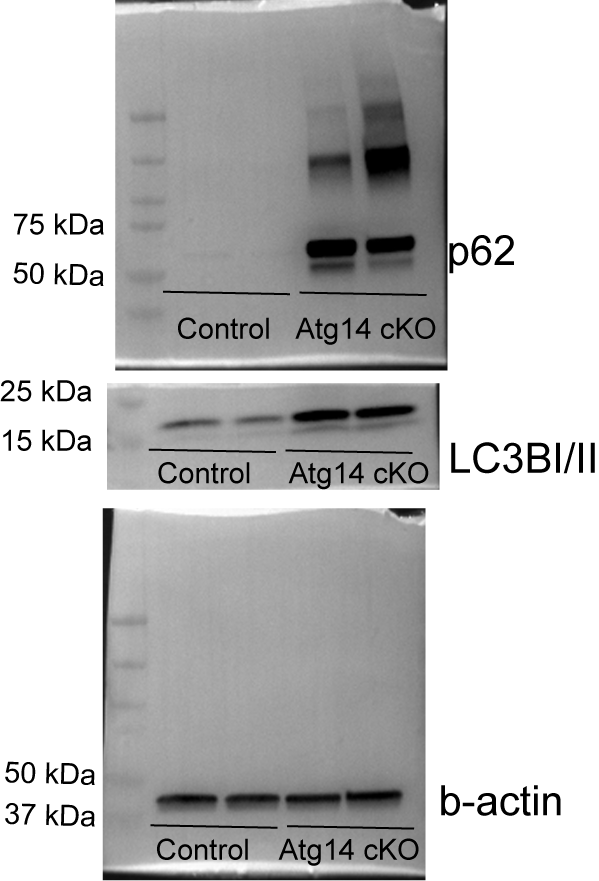

Supplement: Figure 3—figure supplement 2—source data 2. [file elife-97325-fig3-figsupp2-data2.zip › Figure 3-figure supplement 2-source data 1 uncropped original labelled blots/Source data file for supplement figure 3.tif]

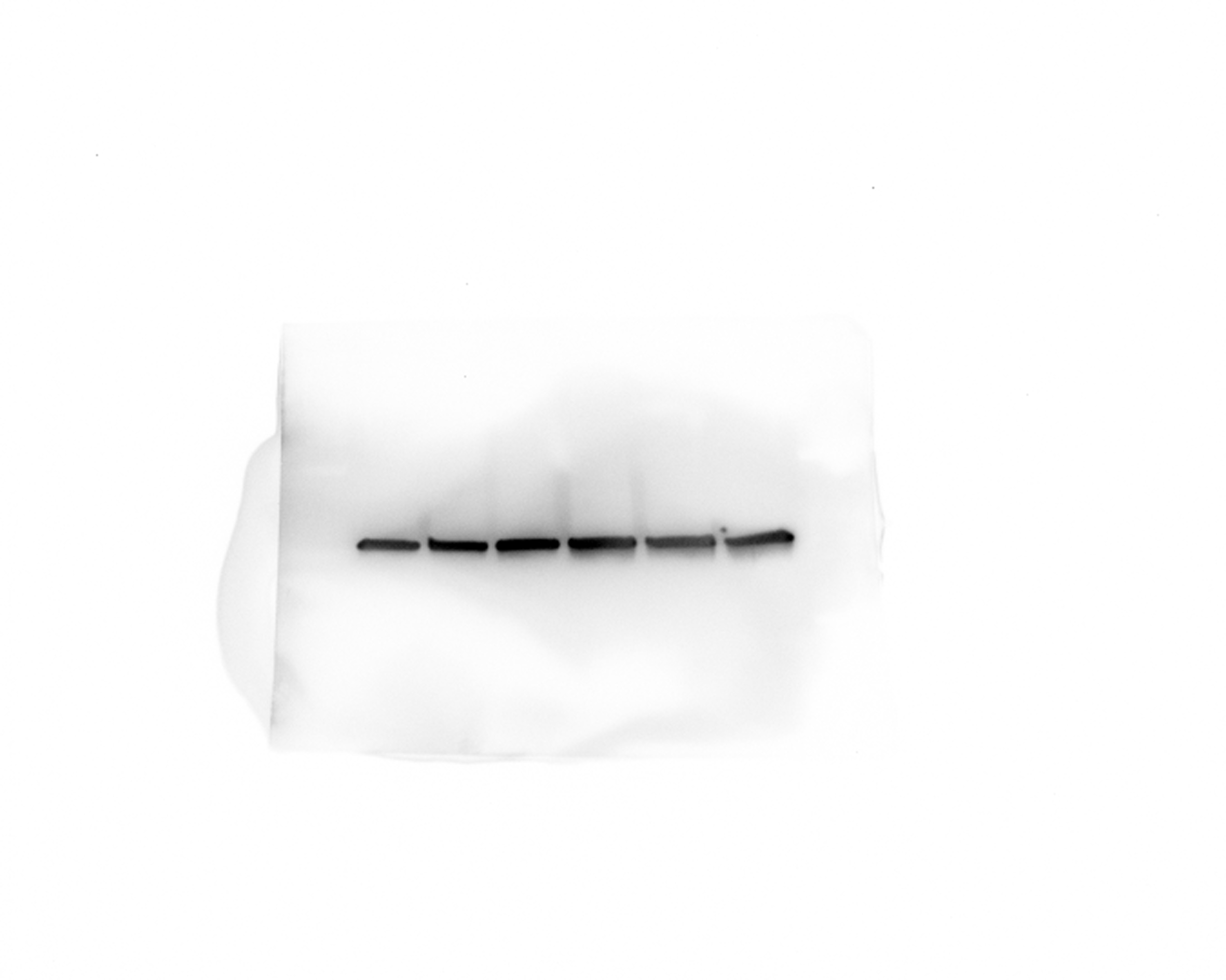

Supplement: Figure 5—source data 1. [file elife-97325-fig5-data1.zip › Uncropped and unedited raw western blot file/b-actin.jpg]

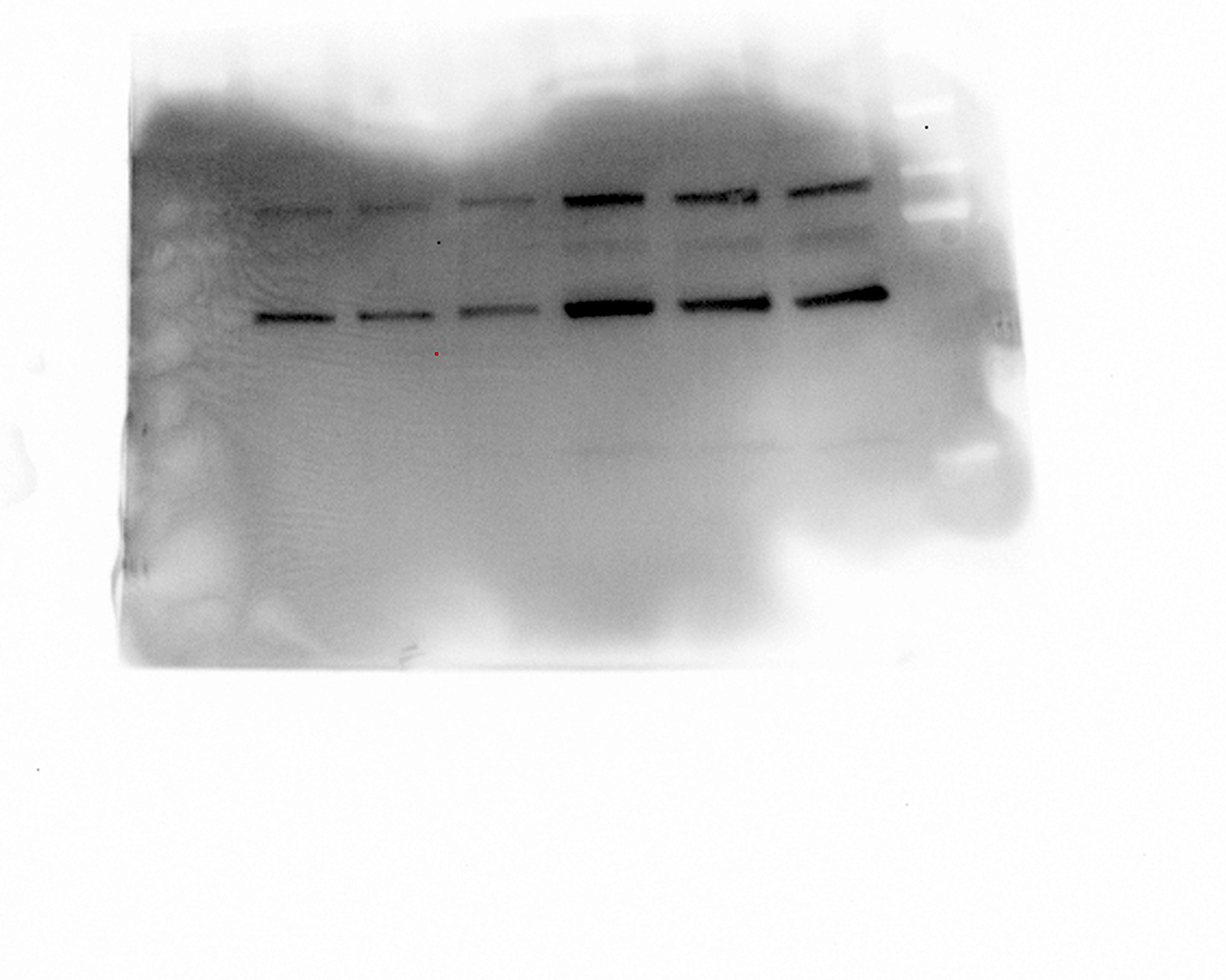

Supplement: Figure 5—source data 1. [file elife-97325-fig5-data1.zip › Uncropped and unedited raw western blot file/caspase-1.jpg]

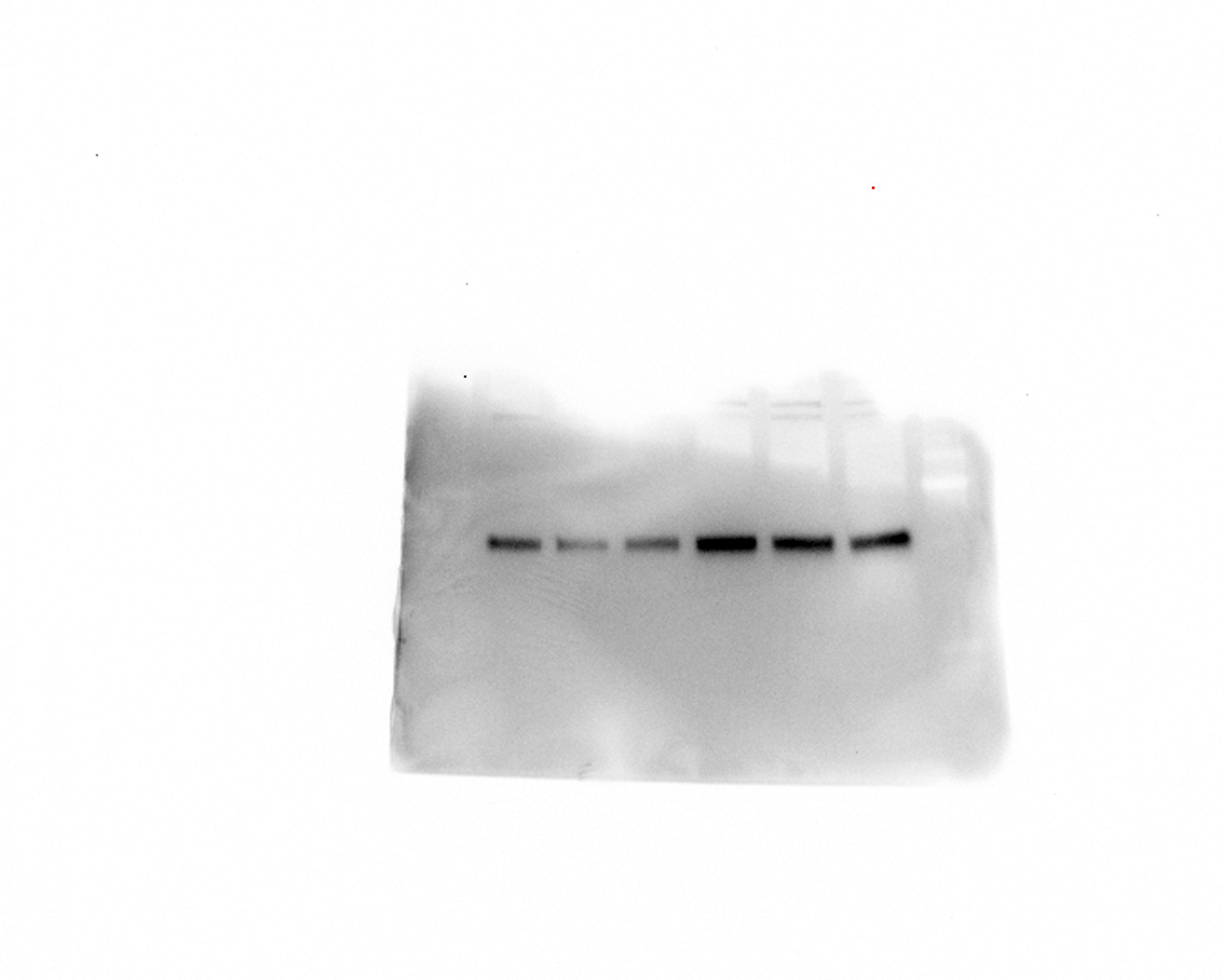

Supplement: Figure 5—source data 1. [file elife-97325-fig5-data1.zip › Uncropped and unedited raw western blot file/gsdmd.jpg]

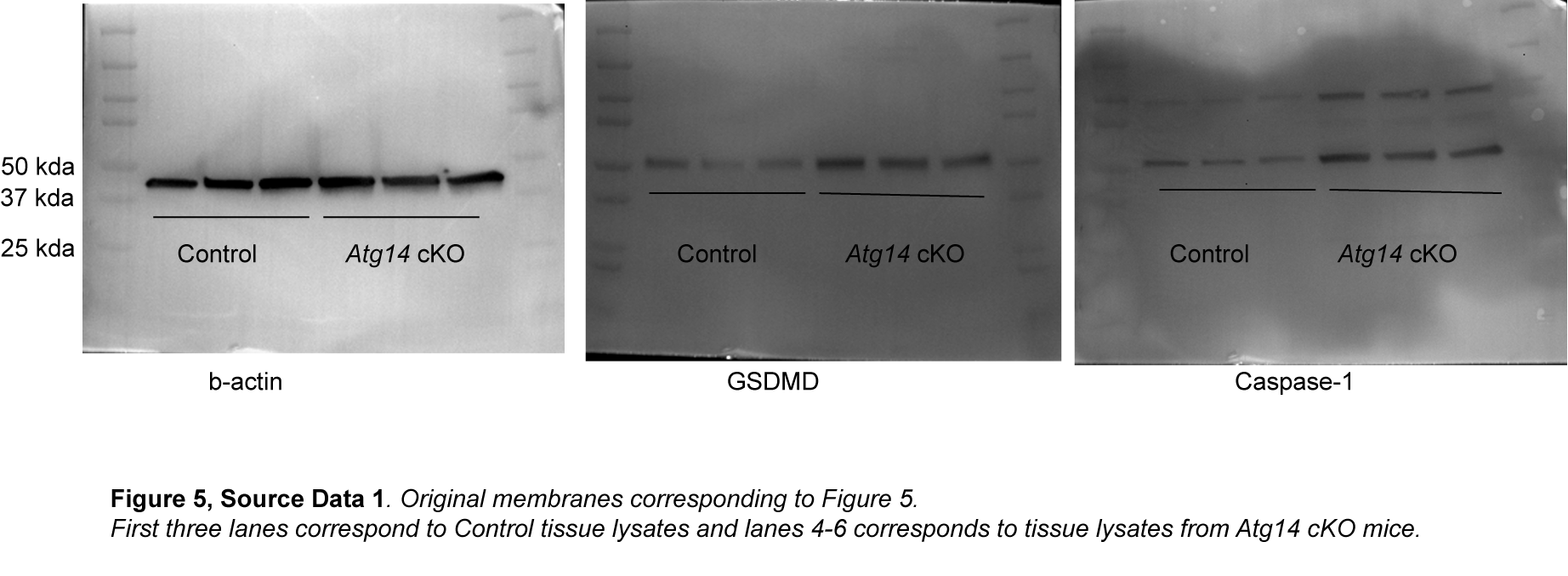

Supplement: Figure 5—source data 2. [file elife-97325-fig5-data2.zip › Uncropped labelled raw western blots/Figure 5 source data file 1.tif]
