# Supplementary material for: The autophagy protein ATG14 safeguards against unscheduled pyroptosis activation to enable embryo transport during early pregnancy
Source: eLife. 2025 Mar 18;13:RP97325. doi: 10.7554/eLife.97325 (PMC11919251; doi:10.7554/eLife.97325)
Supplement: Supplementary file 1. [file elife-97325-supp1.docx]

**Supplementary file 1. List of primers and TaqMan probes**

| **Gene name** | **Species** | **Application, Chemistry** | **Company** | **Sequence/Cat. No.** |
| --- | --- | --- | --- | --- |
| Atg14 f/f | Mouse | Genotyping | IDT | P1: TTGACCGTCACAGGGTGTGAGTGACTT  P2: AAGCAGAGTTAGGCTTCCCTGGTAGAA  P3: CCCATCTCCATTCCTGGATTACTGGAC  P4: CTAAAGCGCATGCTCCAGACTGCCTTG |
| PR^Cre^ | Mouse | Genotyping | IDT | P1: ATGTTTAGCTGGCCCAAA TG  P2: TAT ACC GAT CTC CCT GGA CG  P3: CCC AAA GAG ACA CCA GGA AG |
| Foxj1^Cre^ | Mouse | Genotyping | IDT | P1: ATTTGGGCCAGCTAAACATGC  P2: GCAAAACAGGTAGTTATTCGG |
| *Esr1* | Mouse | qPCR, TaqMan | ABI | Mm00433149_m1 |
| *Pgr* | Mouse | qPCR, TaqMan | ABI | Mm00435628_m1 |
| *Atg14* | Mouse | qPCR, TaqMan | ABI | Mm00553733_m1 |
| *Tnf-alpha* | Mouse | qPCR, TaqMan | ABI | Mm00443258_m1 |
| *Cxcr3* | Mouse | qPCR, TaqMan | ABI | Mm99999054_s1 |
| *Lif* | Mouse | qPCR, TaqMan | ABI | Mm00434761_m1 |
| *Mcm2* | Mouse | qPCR, TaqMan | ABI | Mm00484804_m1 |
| *Ccnd1* | Mouse | qPCR, TaqMan | ABI | Mm00432359_m1 |
| *Fgf18* | Mouse | qPCR, TaqMan | ABI | Mm00433286_m1 |
| *ATG14* | Human | qPCR, TaqMan | ABI | Hs00208732_m1 |
| *18S* | HumanMouse | qPCR, TaqMan | ABI | 4318839 |

*All primer sequences are written 5’ to 3’

ABI-applied biosystems

IDT-integrated DNA technologies.
