# Supplementary material for: The autophagy protein ATG14 safeguards against unscheduled pyroptosis activation to enable embryo transport during early pregnancy
Source: eLife. 2025 Mar 18;13:RP97325. doi: 10.7554/eLife.97325 (PMC11919251; doi:10.7554/eLife.97325)
Supplement: Supplementary file 2. [file elife-97325-supp2.docx]

**Supplementary File 2. List of antibodies**

| **Antibody** | **Company, Catalogue number, and RRIDs** | **Application** |
| --- | --- | --- |
| ATG14 | Proteintech, 24412-1-AP; RRID: AB_2879531 | Immunofluorescence |
| GSDMD | Thermo Fisher Scientific, Cat# PA5-115330; RRID: AB_2899966 | Immunoblotting |
| GSDMD | Abcam, ab209845; PRID: AB_2783550 | Immunofluorescence |
| CASPASE 1 | Abcam, ab138483; RRID: AB_2888675 | Immunoblotting |
| Ki-67 | Abcam, ab15580; RRID: AB_443209 | Immunofluorescence |
| FOXJ1 | Sigma, HPA 005714 | Immunohistochemistry |
| PAX8 | CST, #59019s | Immunohistochemistry |
| MUC1 | Abcam, ab15481; RRID:AB_301891 | Immunofluorescence |
| TOM20 | ab186735; RRID: AB_2889972 | Immunofluorescence |
| CYTOCHROME C (6H2.B4) | Thermo Fisher Scientific, 33-8200; RRID: AB_2533141 | Immunofluorescence |
| Alpha-smooth muscle actin (KRT8) | Developmental Studies Hybridoma Bank, TROMA-I | Immunofluorescence |
| Normal Rabbit IgG | CST, #2729 | Immunofluorescence |
| Goat anti-Rat IgG (H+L) Cross-Adsorbed Secondary Antibody, Alexa Fluor™ 488 | Thermofisher Scientific, A11006;  RRID: AB_2534074 | Immunofluorescence |
| Goat anti-Rabbit IgG (H+L) Highly Cross-Adsorbed Secondary Antibody, Alexa Fluor™ 488 | Thermofisher Scientific, A11034; AB_2576217 | Immunofluorescence |
| Goat anti-Rabbit IgG (H+L) Highly Cross-Adsorbed Secondary Antibody, Alexa Fluor™ 594 | Thermofisher Scientific, A11037;  AB_2534095 | Immunofluorescence |
| GAPDH | CST, #2118S; RRID: AB_561053 | Immunoblotting |
| Anti-rabbit IgG, HRP-linked | CST, #7074 | Immunoblotting |
